# Front-of-Package Protein Labels on Cereal Create Health Halos

**DOI:** 10.3390/foods13081139

**Published:** 2024-04-09

**Authors:** Gina Pope McKeon, William K. Hallman

**Affiliations:** 1 Department of Nutritional Sciences, Rutgers, The State University of New Jersey, New Brunswick, NJ 08901-2882, USA; 2 Department of Human Ecology, Rutgers, The State University of New Jersey, New Brunswick, NJ 08901-2882, USA

**Keywords:** front-of-package labeling, health halo, breakfast cereals, nutrient content claims, food marketing, protein labels

## Abstract

Front-of-package protein labels are frequently added to breakfast cereals, aimed at increasing purchases by consumers who believe they would benefit from eating more protein. However, the overall nutritional compositions of such products are often not significantly better than similar products without protein labels, and may contain more sugar, sodium, and calories to improve taste. We conducted an online survey with 1022 US adults to examine consumer perceptions of two cereals (Special K Original and Special K Protein). Participants perceived Special K Protein as healthier and more nutritious, though less tasty, than Special K Original. Special K Protein was perceived as providing greater health benefits, such as being more likely to help them build muscle, stay healthy, and live longer. Many participants perceived no differences in the amounts of certain nutrients between the cereals, such as sugar (54.5%), sodium (59.2%), and calories (49.1%). Yet, when serving sizes are equalized to one cup, Special K Protein has more sugar, sodium, and calories than Special K Original. Though most participants reported viewing the Facts Up Front label, only 21.3% correctly chose Special K Original as having the larger serving size. This pattern of results suggests the presence of a health halo surrounding the protein-labeled product.

## 1. Introduction

Halo effects, or “health halos”, have been used to describe the illusion that occurs when a product is perceived as healthy overall, based on the presence of a few salient traits rather than on an evaluation of the characteristics of the product in its entirety [1]. For example, products with reduced fat claims are often seen as healthy, even when they contain high levels of sugar and calories [2,3]. Perceiving food as healthy can lead to increased consumption of the product, and to decreased guilt when doing so [4,5]. Perceived healthiness can also affect subsequent food choices and food intake, as the consumption of a food perceived as healthy can influence people to consume more food later [6]. Even the mere presence of foods perceived as healthy as an option can lead individuals to choose to eat more indulgent foods instead [7].

Though research is limited, particular characteristics of product names and titles have been shown to influence perceptions of healthfulness, leading to perceived health halos [5,8,9]. A study of health halo effects from protein-labeled bars found that products with “protein” in their product title led consumers to perceive the product as being more healthful compared to the control bar that did not have “protein” in its title [8]. To expand upon this research and extend it to other types of protein-labeled products, the present study takes advantage of a natural experiment to explore whether a cereal product with the word “protein” in its name elicits the same type of healthfulness perceptions from consumers, leading to a health halo for products that may not be particularly healthy.

For many consumers, the focus is on individual nutrients, either within one food or from their diet as a whole [10]. This, in conjunction with health halos, leads to people overlooking the less healthful characteristics of food products, like increased calories, sugar, and/or fat, in favor of more healthful ingredients, such as fiber or vitamins, which may then result in unhealthy food choices [9,11,12].

Protein is a nutrient that is often a focus for consumers as they search for ways to improve their health through diet, which also frequently results in health halos [10]. Evidence suggests that consumers know little about protein, but perceive it to be healthier than other nutrients [13]. The average intake of protein by most Americans reaches or exceeds recommendations [14]. However, surveys have shown that 71% of people do not know how much protein they need [15], and 60% of consumers are actively trying to increase their protein intake [16].

### 1.1. Cereal and Protein Claims

Understanding health halos related to protein in cereals is of particular importance. Ready-to-eat cereals are consumed by nearly nine-in-ten Americans [17], and 96% of American consumers purchase a box of cereal during every trip to the grocery store [18], making the potential influence of health halos associated with protein in these products substantial. Cereal by itself is often considered to be a healthy choice, particularly when fortified with ingredients perceived as healthful [11,19,20].

Manufacturers began to market cereals featuring protein to keep up with consumers’ growing preferences for “healthier” foods, and to directly compete with other breakfast options, including eggs and Greek yogurt, seen by consumers as high in protein [21]. Through the use of the online product database Label Insight, our data (February 2021) showed that approximately 20% of cereal and breakfast foods now contain protein claims [22]. These products can be profitable, as consumers are willing to pay more for cereal with protein [23].

Kellogg’s, one of the most frequently purchased cereal brands [23], introduced a “Protein Plus” line of their Special K breakfast cereals in 2012 [24]. Since its initial introduction, several varieties of this Special K Protein cereal (SK-PRO) have been sold in grocery stores alongside its original Special K counterpart (SK-ORIG), inviting comparisons between the two products. We hypothesize that compared to SK-ORIG, SK-PRO will be perceived as healthier.

### 1.2. Front-of-Package Nutrition Information: Facts Up Front

Halo effects can lead to consumers focusing on particular nutrients while perhaps ignoring others. Though a lack of attention to nutrition information by a large portion of consumers is well-documented [25,26], studies show that consumers are more likely to view FOP nutrition information than they are to view the Nutrition Facts Panel (NFP) on the side or back of the packaging [12,27,28]. Therefore, the current work also examines the impact of consumer attention to FOP nutrition information with respect to protein and other nutrients.

**Figure 1:**
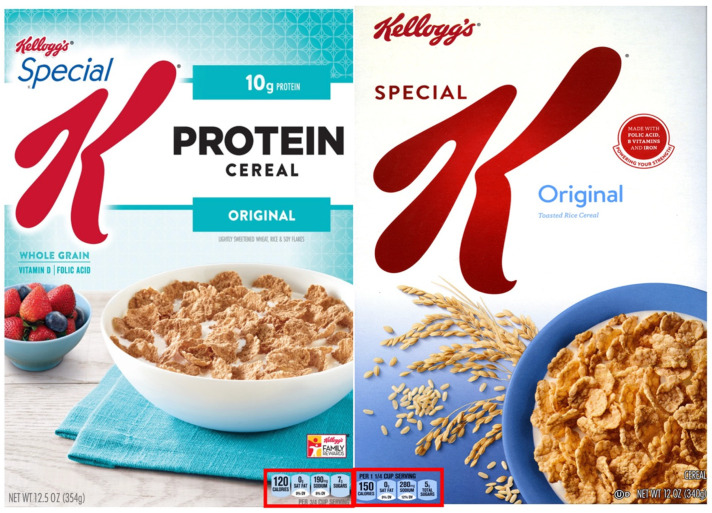
Front-of-package cereal images for Special K Protein and Special K Original used as stimuli in the survey. The Facts Up Front nutrition label is outlined in red.

Facts Up Front (FUF) labels are used by food manufacturers to take select nutrition information from the NFP present on the side or back of the package and to highlight it on the FOP [29,30]. The FUF is designed to provide a concise, easy-to-read summary of some of the nutrients from the NFP, usually including the product’s calorie content and the “nutrients to limit” per serving (e.g., saturated fat, sugar, and sodium). The calories and nutrients in the FUF label are typically displayed in a bubble-like graphic, displayed in a top or bottom corner of the package, with the serving size displayed above or below these “bubbles” [29]. See Figure 1.

In a survey of consumer awareness of FUF labels, ready-to-eat cereal products were most frequently recalled as containing FUF labels [31]. However, the effects of FUF labels on consumer understanding of the nutrient contents of food appear to be mixed. One study that examined consumer nutrient knowledge based on viewing different label conditions found that those who viewed a FUF label scored a higher percentage of correct answers on quizzes that assessed their knowledge of nutrient levels between foods [32]. However, when asked to estimate the level of nutrients in food products, those viewing the FUF were more likely to underestimate the levels of fat and sugars, and to overestimate fiber and protein, compared to the control and other FOP label groups [32].

While FUF labels may invite nutrient comparisons between products, they are not always based on consistent serving sizes. The serving size of SK-PRO is listed as ¾ cup, while for SK-ORIG, it is 1¼ cup, making it difficult for consumers to compare nutrient contents across the two products. To make this potential for misunderstanding clearer, the nutrition information shown on the FOP for each product is listed in Table 1, as well as the same nutrients after equalizing the serving size to one cup. For example, if one were quickly looking at the FUF, without taking notice of the serving sizes, it would appear that SK-ORIG has more calories than SK-PRO (150 calories vs. 120 calories, respectively). However, when the serving sizes of both cereals are equalized, SK-PRO has 160 calories while SK-ORIG has 120 calories per cup. When the serving sizes are based on one cup, SK-PRO contains not just more protein than SK-ORIG, but more of all of the nutrients in the FUF, including more than twice the amount of sugars and 11% more sodium. Because most Americans do not need to increase their intakes of protein, but do need to reduce the amounts of sugar and sodium they consume [14], SK-PRO isn’t necessarily a “healthier” alternative than SK-ORIG. We examine attention to these variations in serving sizes and hypothesize that most participants will be unaware of the differences. Because of the potential for consumers to misinterpret the FUF, we compare the responses of participants who say they looked at the information listed in the FUF with those who say they did not.

### 1.3. Influence of Front-of-Package Nutrition Claims and Purchase Intentions

FOP nutrition claims have also been shown to influence purchase intentions [33]. For example, Verrill et al. [12] demonstrated that a FOP declaration of vitamin fortification (i.e., “Good Source of Calcium and Vitamin D!”) led to increased perceptions of healthfulness and greater purchase intentions. Similarly, the presence of various nutrient content claims on three different products (i.e., yogurt, lasagna, and cereal) influenced study participants to view the product as healthier and to increase purchase intentions [28]. Because purchase intentions can predict actual purchase behaviors [34,35,36], examining them in the context of FOP label claims is valuable.

In this study, we examine whether the FUF label showing less healthful qualities such as sugar and saturated fat is overshadowed by the other more healthful FOP claims, such as protein and fiber. We hypothesize that the SK-PRO cereal will lead to increased perceptions of healthfulness and nutritiousness compared to SK-ORIG. Overall, the main objective of the present study is to examine the effect of a protein label on the perceived nutrient qualities and health benefits of the cereal products. Specifically, we hypothesize that SK-PRO will be perceived as containing more healthful ingredients and fewer less healthful ingredients compared to SK-ORIG. Additionally, we hypothesize that participants, particularly women, will perceive SK-PRO cereal as possessing more health benefits compared to SK-ORIG. We also posit that the presence of a protein label will decrease the perceived tastiness and increase purchase intentions of the cereal. An additional objective of the present research is to investigate consumer attention to and use of FUF labels. In line with the previous research outlined above, we believe attention to these FUF labels will be lacking and will not assist the participants in their ability to answer questions about the nutritional content of each cereal. Lastly, we hypothesize that the presence of FOP claims for protein will lead to increased purchase intentions.

## 2. Materials and Methods

Study participants were recruited from multiple online panels through a market research and online survey software company Qualtrics (www.qualtrics.com (accessed on 20 July 2020)), using quota sampling to reflect the demographics of the population based on 2010 U.S. Census data. Selected panel members (*n* = 8516) were invited to participate in the study via an email invitation, with 4410 of those invited panelists clicking on the link. The final sample included 1022 participants who fully completed the survey and passed attention check questions.

The questionnaire was administered over a 3-week period in August 2018, using the Qualtrics platform. Participants gave informed consent via an online form. The experimental protocol was approved by the Institutional Review Board at Rutgers University (#E15-329). The survey instrument can be found in the Supplementary Materials (S1).

The study was designed to assess consumer perceptions and purchase intentions for two cereals: Special K Original (SK-ORIG), and Special K Protein (SK-Protein). Images of the front of the product packaging of the cereals on the market shelves at the time of the experiment were chosen to represent real-world conditions (See Figure 1 for product stimuli). To control for order effects, the participants viewed the two cereals in random order. In each condition, the participants were first shown an image of the front of the cereal box, followed by corresponding questions about product familiarity, perceived healthfulness, perceived taste, and purchase intention. Participants were then shown the images of the two cereals side-by-side and asked comparative questions about the dependent measures. To reduce participant fatigue and improve survey flow, page breaks were inserted periodically. Corresponding product images were shown after each page break.

Overall, participants were shown images of each product five times. Participants could view the product images while responding to all individual product and comparative questions. However, to prevent them simply looking up the answers, product images displaying the FUF labels were intentionally not visible to the participants when they were asked questions about nutrient amounts and serving sizes. Participants could also not go back to look at the product images. The NFP containing information about nutrients not in the FUF, including total fat, total carbohydrates, and dietary fiber, was never shown to the participants.

This study investigated the impact of a FOP protein claim on multiple health-related outcome measures. Perceived healthfulness, nutritiousness, and tastiness of the control and protein cereals were measured, as well as purchase intentions. Each was measured using 5-point scales (e.g., “How healthy is this product?” with responses ranging from 1 (not healthy at all) to 5 (extremely healthy). Paired samples *t*-tests were performed to determine the differences in the measures between the two products.

Perceived comparative product attributes were measured using 10 items. Participants were asked “Per serving, which of the two products likely has more… (Your best guess is fine)”, for the following: protein, sugar, calories, fiber, whole grains, sodium, Vitamin A, Vitamin D, folic acid, and raisins. Neither product contained raisins. The response option of “No difference” was also available. The items were analyzed as separate dependent variables.

Participants indicated which cereal product was more likely to help them achieve seven health goals: live longer, lose weight, build muscle, feel stronger, stay healthy, have stronger bones, and have a healthier digestive system. None of these health benefits or claims were explicitly displayed on either product. The response options of “No difference” and “I don’t know” were also present. These items were analyzed as separate dependent variables.

Participants chose which cereal they would be more likely to purchase, and which is likely to cost more for the same size package. Willingness to pay was also assessed with the Likert scale question “In comparison to Special K Original, how much more or less would you be willing to pay for Special K Protein?” with responses ranging from 1 (much more) to 7 (much less).

Attention to the FUF nutrition information on the front of the packages was then measured with two items. Participants rated their agreement with the statement “I looked at the nutrition facts information on the front of the boxes to compare them.” Responses were recorded on a Likert scale, ranging from 1 (strongly agree) to 5 (strongly disagree). The choice task asked participants to choose which of the two cereal products had the bigger serving size, with the response options of “No difference” and “I don’t know” also available.

Hypotheses and analyses were specified prior to data collection. All data analyses were conducted using SPSS (Version 26; IBM: Armonk, NY, USA, 2019). Frequencies illustrate to which of the two cereals the participants were more likely to attribute specific nutrients. Multinomial logistic regressions were performed to examine the relationship between participants’ sex and perceived health benefits. Using descriptive statistics, we determined which cereal product was chosen more frequently for the health attribute and nutrient comparison questions. Multinomial logistic regressions were used to examine the relationship between looking at the FUF information and perceived health attributes.

## 3. Results

### 3.1. Participant Demographics

Due to survey recruitment and sampling, participant demographics reflected those of the US population. Of the 1022 participants, 52% were female, the mean age was 47.12 (SD = 16.42), and 65.1% reported doing all of the grocery shopping.

### 3.2. Perceived Healthfulness, Nutritiousness, and Tastiness of Cereals

As shown in Table 2, participants perceived SK-PRO to be healthier and more nutritious, yet less tasty than SK-ORIG. More participants (44.7%) said they were very or extremely familiar with SK-ORIG than with SK-PRO (27.7%).

### 3.3. Perceived Health Benefits

Compared to SK-ORIG, a greater percentage of participants viewed SK-PRO as more likely to help them build muscle, feel stronger, have stronger bones, stay healthy, have healthier digestive systems, lose weight, and live longer (Table 3). Chi-square goodness of fit tests indicated that the distribution was unequal for all health benefits.

Multinomial logistic regressions showed that overall, women, who made up 52% of the sample, were significantly more likely than men to indicate that SK-PRO would be likely to help them achieve all presented health goals as compared to SK-ORIG (Table 4).

### 3.4. Perceived Nutrient Qualities

Though most respondents (79.3%) correctly indicated that SK-PRO had more protein than SK-ORIG, 17.2% said that there was no difference in protein content between the cereals (Table 5). Additionally, 3.5% of participants incorrectly said that SK-ORIG had more protein than SK-PRO. Except for protein, the majority of participants incorrectly indicated that there were no differences between the cereals with respect to all of the other nutrients, based on the displayed serving sizes. Less than a quarter of the participants correctly indicated the SK-PRO had more sugar than SK-ORIG (21.8%), while only 25% correctly chose SK-ORIG as containing more calories and sodium than SK-PRO. However, when serving sizes are equalized to 1-cup, SK-PRO has more calories, sodium, fat, and total carbohydrates and more than twice as much sugar than SK-ORIG (refer to Table 1 for differences). Based on these equalized serving sizes, few participants would be correct in their responses that SK-PRO had more calories, sugar, and sodium compared to SK-ORIG (26.10%, 21.80%, and 15.80%, respectively).

Both cereal products made FOP claims for product attributes and ingredients; as can be seen in Figure 1, SK-PRO had text with the words “WHOLE GRAIN”, “VITAMIN D”, and “FOLIC ACID”, and SK-ORIG had text with the phrase “MADE WITH FOLIC ACID, B VITAMINS, AND IRON”. However, as seen in Table 5, more than half of participants indicated that there were no differences between the products for whole grains, vitamin D, and folic acid (55.5%, 63.0%, and 59.8%, respectively).

Although the amount of fiber was not listed on the FOP of either cereal, one-third (34.4%) of participants reported that SK-PRO contains more fiber than SK-ORIG, while 12.2% chose SK-ORIG as having more fiber than SK-PRO. For the other “healthful” nutrients/qualities of Vitamin A, Vitamin D, folic acid, and whole grains, SK-PRO was more frequently chosen as containing more of these as compared to SK-ORIG.

### 3.5. Attention to Front-of-Package Nutrition Information and Relationship to Perceived Product Attributes

The majority of participants responded that they strongly (29.9%) or somewhat (34.5%) agreed that they looked at the nutrition information located in the FUF on the FOP. However, although the serving sizes for both cereals were displayed with the FOP nutrition information (SK-ORIG = 1¼ cup, SK-PRO = ¾ cup), only 21.3% of participants correctly chose SK-ORIG as having the bigger serving size. The majority of participants incorrectly indicated that there was no difference between cereals (35.8%), that SK-PRO had the bigger serving size (14.8%), or that they did not know (28.1%). 

Table 6 displays the multinomial logistic regressions performed to explore how attention to the FUF affected perceptions of product attributes between cereals (*n* = 844). Those participants who said “neither agree nor disagree” when asked if they looked at the FUF were removed from these analyses (*n* = 178). The categories were also collapsed from a 5-point agreement scale to agree or disagree, where agree is interpreted as “Looked at nutrition facts” and disagree is interpreted as “Did not look at nutrition facts”.

Surprisingly, those who reported looking at the FUF were significantly less likely to correctly indicate that SK-PRO has more protein than SK-ORIG (Table 6). They were also less likely to indicate that SK-PRO has more calories than SK-ORIG, though SK-ORIG had more calories. Moreover, those who reported looking at the FUF were significantly more likely to indicate that SK-PRO has more sugar (the correct response given the values on the FOP) and whole grains. Additionally, those who reported looking at the FUF were significantly less likely to indicate that there was no difference between the two cereals for the nutrients displayed on the FOP: protein, sugar, sodium, and calories, even though there were in fact differences in these nutrients between the cereals. Those who reported looking at the FUF were also significantly less likely to say “I don’t know” when asked which of the two cereals had the bigger serving size. Of those who reported looking at the FUF (*n* = 659), 23.1% correctly identified SK-ORIG as having the bigger serving size as displayed on the FOP. Conversely, 17.0% of those who looked at the FUF incorrectly said that SK-PRO had the bigger serving size, while 34.0% and 25.9% said there was no difference or that they did not know, respectively.

### 3.6. Purchase Intention and Perceptions of Cost

Despite only 19.6% of participants perceiving that SK-PRO would taste better, more than half of all participants (57.3%) indicated that they would be more likely to purchase SK-PRO than SK-ORIG. When asked which of the two cereals is likely to cost more, a majority of participants (59.2%) chose SK-PRO as the more expensive cereal, while 27.9% said there would be no difference in price and 4.6% said SK-ORIG would cost more. Nearly all respondents said that they would be willing to pay about the same (59.6%) or slightly more (24.4%) for SK-PRO compared to SK-ORIG.

## 4. Discussion

Overall, participants in this study perceived SK-PRO to be healthier and more nutritious, though less tasty, than SK-ORIG. Participants also incorrectly attributed health benefits more frequently to the protein-labeled cereal, even though no health benefit claims appeared on the product packaging. Although the protein content declaration of SK-PRO is factually correct, the perception that it is healthier and more nutritious than the original product without a protein claim suggests the existence of a misleading health halo associated with SK-PRO’s protein content. Such health halos may lead consumers to purchase and to pay more for products that they believe offer illusory health benefits, yet may add additional calories, sugars, sodium, and fats to their diets.

That participants perceived SK-PRO to be healthier and less tasty than SK-ORIG is consistent with current literature. Foods with healthier names or descriptions implying that they are healthier are often perceived as being less tasty than their less-healthful counterparts, although this can vary by participant characteristics, such as diet status and food pleasure orientation [5,37,38].

Also consistent with the literature is the seeming inattention to the less healthful ingredients displayed in the FUF, in favor of healthful ingredients [39]. One study examined how a product with FUF labels that displayed both less healthful nutrients, such as saturated fat, and healthful nutrients, such as potassium, were perceived as being more healthful than a product that had only healthful ingredients in the FUF. Additionally, Miller et al. [40] found that although participants did pay attention to the FUFs on two cereal packages, the ability to choose the healthier cereal was low, which is also demonstrated in our results.

Most participants did perceive no differences between cereals with respect to the content of most nutrients displayed. However, of those who did perceive differences, SK-ORIG was chosen as having more of the less healthful nutrients, such as sugar and sodium, while they more frequently chose SK-PRO as having more of the healthful nutrients, such as protein, fiber, folic acid, and Vitamin D.

The percentage of participants who reported viewing the FUF is also consistent with previous studies [27]. However, the inaccuracies in participant responses when asked to indicate nutrient amounts and serving sizes reveal their lack of attention to this FUF information. Those who reported looking at the FUF were generally more accurate in indicating which product had more of certain nutrients per serving but were inaccurate in their perception of which cereal had a bigger serving size. These participants were also less likely to choose “no difference” in nutrient amounts between products, likely because the FUF labels make it obvious that there were differences in the products’ nutritional makeups.

Even though the majority of participants reported that they looked at the FUF, many fewer correctly chose SK-ORIG as having the bigger serving size. This may be because the participants simply did not look closely at the serving sizes located at the top of the FUF graphic, and instead paid closer attention to the nutrient amounts in the main “bubbles” portion of the graphic. Additionally, the intentional unavailability of the product images to participants during questions regarding nutrient amounts allowed us to observe the possibility of a recall bias. As demonstrated here, a biased recall of information may have contributed to the increased perceptions of health for SK-PRO, in which the inability to recall the correct nutrient amount information led participants to rely on other cognitive biases, such as the halo effect.

Although the FOP of SK-ORIG highlighted that it was “Made with Folic Acid, B vitamins and Iron” and “Powering your strength” (albeit in small lettering), only about 5% of the participants believed that SK-ORIG would be more likely than SK-PRO to help them feel stronger (5.6%) and build muscle (4.7%). In contrast, 43.3% thought that SK-PRO would be more likely than SK-ORIG to help them feel stronger, and 54% thought it would help them build muscles. This suggests that the participants consider protein-enriched foods to be functional foods, providing a health benefit beyond their basic nutritional value.

Many participants also attributed other health benefits to the SK-PRO cereal, such as having a healthier digestive system and stronger bones, though these benefits are not associated with protein. This is consistent with the literature, where, when asked to name a food or nutrient that they consider beneficial for a health issue they experience, such as cardiovascular problems, weight loss, and lack of energy, participants most frequently cited protein [41]. This suggests that participants may be aware of some health benefits associated with protein but lack the knowledge of how these benefits apply to their own diets and lifestyles. This is consistent with studies showing that consumers often possess attribute-related knowledge for high-protein and other functional foods, but lack knowledge about the health consequences of consuming them [42]. These results do not align with those of a study of European consumers, which found that people were quite knowledgeable about the function of protein in the body, though the older study participants were skeptical about products that had increased protein content and their effects on health [43]. In contrast, our results seem to portray a misunderstanding or confusion about the role of protein in the body, with many participants indicating that SK-PRO would be more likely to affect health benefits, such as help them live longer or have stronger bones than SK-ORIG.

It is possible that some of the falsely attributed health benefits may be the result of other FOP information on the cereal boxes. For example, although dietary fiber content did not appear on the FOP of either product, the SK-PRO cereal noted that it was made with whole grains, perhaps suggesting to participants that it was fiber-rich, and thus promoted a healthier digestive system. Additionally, for all the questions about comparative health benefits between cereals, more than 10% of participants chose “I don’t know.” This highlights a lack of nutrition, food, and health literacy that exists among many consumers, which can be problematic for food choice and decisions that can impact health [44,45].

Many participants reported that SK-PRO would not taste as good as SK-ORIG but said they would be more likely to purchase SK-PRO and would pay the same or more for it. Our results are aligned with the Unhealthy = Tasty Intuition first defined by Raghunathan, Naylor, and Hoyer [37], in which perceived healthiness and tastiness are inversely related. This may be explained by research that has found that consumers are willing to pay a premium for functional foods or products with health claims [46,47]. Additional research should further explore the relationship between perceived health, tastiness, and willingness to pay.

These increased purchase intentions for SK-PRO may also translate into real-world shopping behaviors. One study found that intentions are more highly correlated with actual purchases for specific, existing products, rather than with new and broadly categorized products, such as generic mock cereals that are often used in studies [34]. Our study utilized two existing cereal products with which many participants were familiar. Thus, the results from this study demonstrate that a protein label on cereal may lead to increased purchase intentions, which could translate to actual purchase behavior in a real-world setting, complementing purely experimental manipulations.

## 5. Conclusions

These results demonstrate that a health halo may surround a product when a protein label is present. A protein label may mislead consumers to believe that a product is healthier, has more healthful attributes and less unhealthy attributes, and is more likely to contribute to positive health benefits than comparable non-protein-labeled counterparts. These consumer misunderstandings in combination with marketing tactics that emphasize protein content have implications for consumer health. Our results indicate the need for clear and simple messaging of protein claims that provides actionable and realistic advice to consumers about how to navigate protein labeling and make healthier food choices. These results provide a foundation for policymakers to review existing labeling regulations, with the goal of making labels more understandable, consistent, and concise. Limiting the number of claims made on the FOP, highlighting less healthful nutrients on the FOP instead of solely displaying those perceived as more healthful, and restricting the use of “protein” in the name of products to those that are excellent or complete sources of protein could support consumers in making wiser choices.

These findings also reveal that consumers may not have a full understanding of serving sizes and how to calculate nutrient content between products when serving sizes differ. This highlights the need for consumer education about the health halo pitfalls of reading FOP labels. Our conclusions could provide nutrition professionals with a better understanding of the protein knowledge consumers may lack, allowing them to tailor their nutrition education they may provide to help clients reach their health goals through informed choices.

## Figures and Tables

**Table 1:** Comparison of nutrition information by actual serving size and equalized serving size between cereals.

Nutrient/Quality	Displayed Serving Size		Equalized Serving Size	
	SK-PRO	SK-ORIG	SK-PRO	SK-ORIG
Serving Size *	¾ cup	1¼ cup	1 cup	1 cup
Calories *	120	150	160	120
Sugars *	7 g	5 g	9.34 g	4 g
Sodium *	190 mg	280 mg	253.34 mg	224 mg
Protein ^±^	10 g	7 g	13.34 g	5.6 g
Total Fat	1 g	0.5 g	1.34 g	0.4 g
Sat Fat *	0 g	0 g	0 g	0 g
Total Carbohydrate	19 g	29 g	25.34 g	23.2 g
Dietary Fiber	3 g	<1 g	4 g	<1 g

SK-PRO = Special K Protein. SK-ORIG = Special K Original. * Present within the Facts Up Front for both SK-PRO and SK-ORIG. ^±^ Present on the Front-of-Package for SK-PRO only. Nutrients without a superscript were not shown on the Front-of-Package for either product.

**Table 2:** Paired *t*-test results for ratings of healthfulness, nutritiousness, tastiness, and likelihood of purchase between cereals.

	Mean	Std Dev	S.E. Mean	Paired *t* Test			
				*t* Value	df	Sig (Two-Tailed)	Cohen’s *d*
Healthfulness							
SK-PRO	3.47	0.985	0.031	2.064	1021	0.039	0.040
SK-ORIG	3.43	1.001	0.031				
Nutritiousness							
SK-PRO	3.46	0.985	0.031	3.281	1021	0.001	0.070
SK-ORIG	3.39	1.004	0.031				
Tastiness							
SK-PRO	3.09	1.159	0.036	−3.682	1021	0.000	0.078
SK-ORIG	3.18	1.156	0.036				
Familiarity							
SK-PRO	2.53	1.399	0.044	−18.335	1021	0.000	0.540
SK-ORIG	3.26	1.305	0.041				
Likely to purchase							
SK-PRO	3.30	1.395	0.044	2.734	1021	0.006	0.071
SK-ORIG	3.20	1.439	0.045				

SK-PRO = Special K Protein, SK-ORIG = Special K Original.

**Table 3:** Chi-square goodness of fit test results for perceived health benefits.

					Chi-Square Goodness of Fit Test		
	Protein	Original	No Difference	I Don’t Know	χ^2^	df	*p*
Build muscle	54.0%	4.7% *	31.3%	10.0%	621.10	3	<0.001
Feel stronger	43.3%	5.6% *	39.1%	11.9%	443.29	3	<0.001
Have stronger bones	37.4%	6.9% *	43.4%	12.2%	401.59	3	<0.001
Stay healthy	29.1%	9.2% *	51.5%	10.3%	438.86	3	<0.001
Have healthier digestive system	27.9%	11.1% *	48.4%	12.6%	370.02	3	<0.001
Lose weight	27.2%	13.6% *	46.4%	12.8%	302.53	3	<0.001
Live longer	20.2%	7.7% *	55.9%	16.2%	552.46	3	<0.001

* *p* < 0.001 A post-hoc binomial test with Bonferroni correction between Special K Original and Special K Protein indicated that there were statistically significant differences in these percentages for all items (*p* < 0.001 for all binomial tests).

**Table 4:** Multinomial logistic regression results for the interaction between participants’ sex and perceived health benefits.

	SK-PRO vs. SK-ORIG		No Difference vs. SK-ORIG		IDK vs. SK-ORIG	
Health Benefit	Odds Ratio	95% CI	Odds Radio	95% CI	Odds Radio	95% CI
Lose weight						
Female	2.216 ***	1.455–3.374	2.232 ***	1.508–3.303	2.460 ***	1.506–4.019
(Male)						
Build muscle						
Female	2.544 **	1.351–4.790	2.493 **	1.303–4.770	2.574 **	1.248–5.310
(Male)						
Feel stronger						
Female	2.428 **	1.336–4.413	3.025 ***	1.659–5.517	3.063 ***	1.566–5.992
(Male)						
Stay healthy						
Female	2.406 ***	1.479–3.915	2.385 ***	1.501–3.789	2.710 ***	1.520–4.831
(Male)						
Live longer						
Female	2.120 **	1.221–3.682	2.840 ***	1.710–4.715	3.222 ***	1.821–5.698
(Male)						
Have stronger bones						
Female	1.791 *	1.065–3.011	1.866 *	1.116–3.120	2.214 **	1.220–4.018
(Male)						
Have a healthier digestive system						
Female	2.231 ***	1.419–3.507	2.258 ***	1.475–3.459	2.553 ***	1.515–4.301
(Male)						

SK-PRO = Special K Protein, SK-ORIG = Special K Original, IDK = I don’t know. All health benefits were treated as separate dependent variables. *n* = 1022, Reference category is SK-ORIG. * *p* < 0.05, ** *p* < 0.01, *** *p* < 0.001.

**Table 5:** Chi-square goodness of fit tests results for participant perceptions of which cereal contains more of a given nutrient/quality.

				Chi-Square Goodness of Fit Test		
	SK-PRO	SK-ORIG	No Difference	χ^2^	df	*p*
Protein ^†^	**79.3%**	3.5%	17.2%	998.66	2	<0.001
Fiber	**34.3%**	12.2%	53.4%	260.61	2	<0.001
Whole grains ^†^	31.5%	13.0%	55.5%	277.99	2	<0.001
Calories ^†‡^	26.1% ^a^	**24.8% ^a^**	49.1%	114.89	2	<0.001
Vitamin D ^†^	25.4%	11.5%	**63.0%**	434.73	2	<0.001
Folic Acid ^†‡^	25.0%	**15.2%**	59.8%	336.75	2	<0.001
Sugar ^†‡^	**21.8% ^b^**	23.7% ^b^	54.5%	206.60	2	<0.001
Vitamin A	19.8%	11.4%	**68.8%**	588.67	2	<0.001
Sodium ^†‡^	15.8%	**25.0%**	59.2%	320.91	2	<0.001
Raisins *	12.1% ^cd^	8.9% ^c^	**79.0% ^d^**	959.13	2	<0.001

SK-PRO = Special K Protein, SK-ORIG = Special K Original. Post-hoc binomial pairwise tests with Bonferroni correction were performed between SK-PRO, SK-ORIG, and No difference responses for all product attribute frequencies. Bolded text indicates correct responses based on nutrient amounts per displayed serving size. * Neither product contained raisins. ^†^ These nutrients were displayed on the front-of-package or in the Facts Up Front for SK-PRO. ^‡^ These nutrients were displayed on the front-of-package or in the Facts Up Front for SK-ORIG. Means with the same superscript letter are not significantly different from each other at *p* < 0.05.

**Table 6:** Multinomial logistic regression results for the interaction between looking at facts up front and perceived product ingredients and nutrients.

	SK-PRO vs. SK-ORIG		No Difference vs. SK-ORIG		IDK vs. SK-ORIG	
Ingredient/Nutrient	Odds Ratio	95% CI	Odds Ratio	95% CI	Odds Ratio	95% CI
Protein						
Looked at FUF	0.134 *	0.018–0.992	0.069 **	0.009–0.523		
(Did not look at FUF)						
Sugar						
Looked at FUF	2.080 *	1.121–3.859	0.438 ***	0.288–0.666		
(Did not look at FUF)						
Sodium						
Looked at FUF	0.730	0.392–1.360	0.305 ***	0.193–0.484		
(Did not look at FUF)						
Calories						
Looked at FUF	0.575 *	0.345–0.961	0.347 ***	0.221–0.545		
(Did not look at FUF)						
Fiber						
Looked at FUF	1.294	0.725–2.310	0.635	0.373–1.079		
(Did not look at FUF)						
Vitamin A						
Looked at FUF	1.187	0.614–2.293	0.655	0.380–1.128		
(Did not look at FUF)						
Vitamin D						
Looked at FUF	1.324	0.693–2.532	0.567 *	0.325–0.990		
(Did not look at FUF)						
Folic acid						
Looked at FUF	1.361	0.738–2.512	0.508 **	0.306–0.843		
(Did not look at FUF)						
Whole grains						
Looked at FUF	1.942 *	1.083–3.482	0.698	0.421–1.155		
(Did not look at FUF)						
Raisins						
Looked at FUF	1.014	0.387–2.658	0.366 **	0.172–0.778		
(Did not look at FUF)						
Bigger serving size ^†^						
Looked at FUF	1.125	0.600–2.107	0.628	0.388–1.017	0.473 **	0.291–0.768
(Did not look at FUF)						

FUF = Facts Up Front, SK-PRO = Special K Protein, SK-ORIG = Special K Original, IDK = I don’t know. All product attributes were treated as separate dependent variables. *n* = 844, Reference category is SK-ORIG. * *p* < 0.05, ** *p* < 0.01, *** *p* < 0.001. ^†^ This was a separate question with “I don’t know” as an additional response option. OR represent Odds Ratios for those who reported looking at the FUF vs. those who reported not looking at the FUF.

## Data Availability

The original contributions presented in the study are included in the article and Supplementary Materials, further inquiries can be directed to the corresponding authors.

## Supplementary Materials

The following supporting information can be downloaded at: https://www.mdpi.com/article/10.3390/foods13081139/s1, S1. Survey Instrument.
