# Supplementary material for: Front-of-Package Protein Labels on Cereal Create Health Halos
Source: Foods. 2024 Apr 9;13(8):1139. doi: 10.3390/foods13081139 (PMC11049005; doi:10.3390/foods13081139)
Supplement: Supplementary file 1 [file foods-13-01139-s001.zip › foods-2912870-supplementary.pdf]

S1. Survey Instrument

(The informed consent was shown to participants here.)

Do you agree to participate in this study?

○ Yes.

○ No.

In which state do you currently reside?

▼ Alabama ... Wyoming

What is your age?

____

What is your gender?

○ Male

○ Female

End of Block: Consent + Quota Screener

Start of Block: Part I: Intro

Start of Block: Control Special K

(The stimuli of the SK-ORIG cereal image was shown here.)

Please answer the following questions about the product shown above.

How familiar are you with this product?

- ◯ Not familiar at all
- ◯ Slightly familiar
- ◯ Moderately familiar
- ◯ Very familiar
- ◯ Extremely familiar

How often have you eaten this product in the last year?

- ◯ Never
- ◯ A few times
- ◯ Often
- ◯ Very often
- ◯ Extremely often

How healthy is this product?

- ◯ Not healthy at all
- ◯ Slightly healthy
- ◯ Moderately healthy
- ◯ Very healthy
- ◯ Extremely healthy

How nutritious is this product?

- Not nutritious at all
- Slightly nutritious
- Moderately nutritious
- Very nutritious
- Extremely nutritious

Page Break

(The stimuli of the SK-ORIG cereal image was shown here again.)

How good do you think this product tastes?

- Not good at all
- Somewhat good
- Moderately Good
- Very Good
- Extremely good

How likely would you be to purchase this product in the next 6 months?

- Extremely likely
- Somewhat likely
- Neither likely nor unlikely
- Somewhat unlikely
- Extremely unlikely

End of Block: Control Special K

Start of Block: Protein Special K

(The stimuli of the SK-PRO cereal image was shown here.)

Please answer the following questions about the product shown above.

How familiar are you with this product?

- Not familiar at all
- Slightly familiar
- Moderately familiar
- Very familiar
- Extremely familiar

How often have you eaten this product in the last year?

- ◯ Never
- ◯ A few times
- ◯ Often
- ◯ Very often
- ◯ Extremely often

How healthy is this product?

- ◯ Not healthy at all
- ◯ Slightly healthy
- ◯ Moderately healthy
- ◯ Very healthy
- ◯ Extremely healthy

How nutritious is this product?

- ◯ Not nutritious at all
- ◯ Slightly nutritious
- ◯ Moderately nutritious
- ◯ Very nutritious
- ◯ Extremely nutritious

Page Break

(The stimuli of the SK-PRO cereal image was shown here again.)

How good do you think this product tastes?

- ◯ Not good at all
- ◯ Somewhat good
- ◯ Moderately Good
- ◯ Very Good
- ◯ Extremely good

How likely would you be to purchase this product in the next 6 months?

- ◯ Extremely likely
- ◯ Somewhat likely
- ◯ Neither likely nor unlikely
- ◯ Somewhat unlikely
- ◯ Extremely unlikely

End of Block: Protein Special K

Start of Block: Comparison Questions

After looking at the pictures of the two boxes of cereal below, please answer the questions that follow.

(The two product images were shown side-by-side here.)

If you saw these two boxes of cereal on the shelf, which would you be more likely to purchase?

- ○ Special K Original
- ○ Special K Protein

Why would you be more likely to purchase this cereal?

____

Which of these two cereals would most likely taste best?

- ○ Special K Original
- ○ Special K Protein
- ○ No difference
- ○ I don't know

Page Break

(The product images were shown again.)

Which of these two cereals would be most likely to help you...

	Special K Original	Special K Protein	No difference	I don't know
Lose weight?	○	○	○	○
Build muscle?	○	○	○	○
Feel stronger?	○	○	○	○
Stay healthy?	○	○	○	○
Live longer?	○	○	○	○
Have stronger bones?	○	○	○	○
Have a healthier digestive system?	○	○	○	○

Page Break

(The product images were shown again.)

Which of these two cereals is likely to cost more for the same size box of cereal?

○ Special K Original

○ Special K Protein

○ No difference

○ I don't know

In comparison to Special K Original, how much more or less would you be willing to pay for Special K Protein?

- Much more
- Moderately more
- Slightly more
- About the same
- Slightly less
- Moderately less
- Much less

Page Break

Per serving, which of the two products likely has more... (Your best guess is fine.)

	Special K Original	Special K Protein	No difference
Protein?	○	○	○
Sugar?	○	○	○
Sodium?	○	○	○
Calories?	○	○	○
Fiber?	○	○	○
Vitamin A?	○	○	○
Vitamin D?	○	○	○
Folic Acid?	○	○	○
Whole grains?	○	○	○
Raisins?	○	○	○

Page Break

I looked at the nutrition facts information on the front of the boxes to compare them.

- Strongly agree
- Somewhat agree
- Neither agree nor disagree
- Somewhat disagree
- Strongly disagree

Which of the two products had the bigger serving size? (Your best guess is fine.)

- Special K Original
- Special K Protein
- No difference
- I don't know.

End of Block: Comparison Questions

Start of Block: Attention Check

Do you disagree or agree with the following statement: "I read the material in this study carefully"?

- Completely disagree 1
- Somewhat disagree 2
- Neither disagree nor agree 3
- Somewhat agree 4
- Completely agree 5

Do you disagree or agree with the following statement: "I rushed through answering the questions in this study"?

- Completely disagree 1
- Somewhat disagree 2
- Neither disagree nor agree 3
- Somewhat agree 4
- Completely agree 5

End of Block: Attention Check

Start of Block: Demographics

Education (Highest Degree Received)

- Less than high school
- High school diploma or GED
- Some college
- Bachelor's degree
- Post graduate
- Prefer not to answer

Which of the following best describes you?

Select all boxes that apply. Note, you may select more then one group.

☐ **White** For example, German, Irish, English, Italian, Polish, French, etc.

☐ **Hispanic, Latino, or Spanish origin** For example, Mexican or Mexican American, Puerto Rican, Cuban, Salvadoran, Dominican, Colombian, etc.

☐ **Black or African Am.** For example, African American, Jamaican, Hitian, Nigerian, Ethiopian, Somalian, etc.

☐ **Asian** For example, Chinese, Filipino, Asian Indian, Vietnamese, Korean, Japanese, etc.

☐ **American Indian or Alaska Native** For example, Navajo Nation, Blackfeet Tribe, Mayan, Aztec, Native Village of Barrow Inupiat Traditional Government, Nome Eskimo Community, etc.

☐ **Middle Eastern or North African** For example, Lebanese, Iranian, Egyptian, Syrian, Moroccan, Algerian, etc.

☐ **Native Hawaiian or Other Pacific Islander** For example, Native Hawaiian, Samoan, Chamorro, Tongan, Fijian, Marshallese, etc.

☐ **Some other race, ethnicity, or origin.**

☐ Prefer not to answer.

Number of people living in household (including yourself):

____

Do you have any child under 18 live in your household?

○ Yes

○ No

○ Prefer not to answer

Household income:

- Less than $25,000
- $25,000 to $49,999
- $50,000 to $99,999
- $100,000 or more
- Prefer not to answer

Marital status

- Single, never married
- Married
- Widowed
- Divorced
- Separated
- Living with partner
- Prefer not to answer

When it comes to grocery shopping for your household:

- You do all of it
- You do most of it
- You do about half of it
- Someone else does most of it, you do some of it
- Someone else does all of it
- Prefer not to answer

Q177 What is your native language/mother tongue?

- English
- Spanish
- Other, specify ____

End of Block: Demographics
